# Discrimination, Depression, and Anxiety Among US Adults

**DOI:** 10.1001/jamanetworkopen.2025.2404

**Published:** 2025-03-28

**Authors:** Monica L. Wang, Marie-Rachelle Narcisse

**Affiliations:** 1 Department of Community Health Sciences, Boston University School of Public Health, Boston, Massachusetts; 2 Department of Health Policy and Management, Harvard T.H. Chan School of Public Health, Boston, Massachusetts; 3 Department of Psychiatry and Human Behavior, Brown University, Providence, Rhode Island

## Abstract

**Importance:**

Examining how discrimination is associated with mental health across different demographic groups can guide efforts to improve mental well-being.

**Objectives:**

To analyze associations between discrimination and mental health and explore how these associations may vary by race and ethnicity and sex.

**Design, Setting, and Participants:**

Cross-sectional data of 29 522 adults weighted to represent a population of 258 237 552 US adults from the 2023 US National Health Interview Survey were analyzed.

**Exposures:**

Exposure to discrimination was measured using the Everyday Discrimination Scale, which assesses frequency of being treated with less courtesy, receiving poor service, being treated as not smart, being feared, and experiencing harassment. A summative scale and a nominal variable (none, low, and high) measured degree and levels of discrimination exposure.

**Main Outcomes and Measures:**

Outcomes included depression (measured with the Patient Health Questionnaire-2 scale) and anxiety (measured with the Generalized Anxiety Disorder-2 scale), with scores of 3 or greater indicating positive screening results for each scale. Multinomial logistic regression analyses examined associations of interest and tests of interaction explored effect modification by race and ethnicity and sex.

**Results:**

Among a sample of 29 522 adults weighted to represent a population of 258 237 552 US adults (mean age, 48.1 years [95% CI, 47.8-48.4 years]; 51.1% female; 17.5% Hispanic or Latino, 2.5% multiracial or other, 6.2% non-Hispanic Asian, 11.6% non-Hispanic or non-Latino Black, and 62.2% non-Hispanic or non-Latino White), each unit increase in exposure to discrimination was associated with increased odds of positive screening results for depression (odds ratio [OR], 1.15 [95% CI, 1.12-1.17]), anxiety (OR, 1.14 [95% CI, 1.12-1.16]), and both depression and anxiety (OR, 1.19 [95% CI, 1.16-1.21]). Compared with no exposure to discrimination, low and high exposure to discrimination were associated with increased odds of positive screening results for depression (low exposure: OR, 2.20 [95% CI, 1.77-2.72]; high exposure: OR, 5.39 [95% CI, 3.61-8.04]), anxiety (low exposure: OR, 1.97 [95% CI, 1.66-2.33]; high exposure: OR, 4.98 [95% CI, 3.59-6.91]), and both depression and anxiety (low exposure: OR, 2.60 [95% CI, 2.13-3.18]; high exposure: OR, 8.84 [95% CI, 6.44-12.14]). Associations between discrimination and positive screening results for depression alone (*F*_4,607_ = 3.35; *P* = .01) and between discrimination and positive screening results for both depression and anxiety (*F*_4,607_ = 2.80; *P* = .03) varied by race and ethnicity. Associations of interest did not differ by sex.

**Conclusions and Relevance:**

Findings of this cross-sectional study suggest an association between discrimination and mental health across US adults, emphasizing the need for further evaluation and increased awareness of how these associations may vary across different demographic groups.

## Key Points

**Question:**

What is the association of discrimination with mental health among US adults across different demographic groups?

**Findings:**

In this cross-sectional study using data from the US National Health Interview Survey, which includes a nationally representative sample of 29 522 US adults weighted to represent a population of 258 237 552 US adults, higher exposure to discrimination was significantly associated with increased odds of positive screening results for anxiety, depression, and both anxiety and depression. Associations between discrimination and screening positive for depression and anxiety varied by race and ethnicity, but not by sex.

**Meaning:**

Findings suggest the need to raise awareness of the association between discrimination and mental health across demographic groups, as well as the importance of further mental health evaluation to promote mental well-being and address disparities.

## Teaser

This cross-sectional study uses data from the US National Health Interview Survey to analyze associations between discrimination and mental health and explore how these associations may vary by race and ethnicity and sex.

## Introduction

Discrimination is increasingly recognized for its effect on physical and mental health.^1,2,3^ Everyday discrimination refers to routine, often subtle, forms of mistreatment that individuals experience, such as being treated with less respect, encountering microaggressions, or receiving inferior service.^4^ These interpersonal interactions are often based on negative stereotypes that others hold about an individual’s identity or background. Discrimination is reinforced and upheld by structural and systemic factors,^5^ with 31% of US adults having experienced at least one major incident of discrimination in their lifetime (eg, denied a job or promotion for unjust reasons, being prevented from moving into a neighborhood due to discriminatory actions) and 63% encountering discrimination daily.^6,7^

Chronic exposure to discrimination increases the risk of psychological distress, depression, and anxiety,^1,8,9^ as well as conditions such as hypertension and cardiovascular disease.^2,10,11^ Adverse health associations of discrimination are most pronounced among marginalized groups including women; Asian, Black, Hispanic or Latino, Indigenous, and multiracial individuals; those with lower socioeconomic status; LGBTQ+ (lesbian, gay, bisexual, transgender, or queer) individuals; and those with disabilities.^12,13,14^ These groups face disproportionate health burdens due to discrimination and structural barriers to health care and healthy living conditions.^15,16^ Longitudinal studies reveal that discrimination is associated with increased risk of developing depressive symptoms and anxiety over time^17,18,19^ as well as racial and ethnic mental health disparities.^20^

Depression and anxiety continue to be critical concerns in the US. Depression rates among women increased from 10% in 2013-2016 to 23% in 2023, while rates among men similarly increased from 5.5% to 22%.^21,22^ Anxiety rates among women increased from 8% to 31% and among men from 5% to 24% from 2018 to 2023.^22,23^ Among Black adults, rates of depression increased from 9% in 2013-2016 to 21% in 2023 and rates of anxiety increased from 6% in 2018 to 27% in 2023; among Hispanic or Latino adults, rates of depression increased from 8% to 28% and rates of anxiety increased from 6% to 32%; and among White adults, rates of depression increased from 9% to 21% and rates of anxiety increased from 7% to 27%.^21,22,23^

Given these trends, it is important to investigate how discrimination is associated with mental health across a representative range of demographic groups. Previous research on the association between discrimination and mental health has often been limited by smaller sample sizes or focused primarily on comparisons between Black and White populations or between Hispanic or Latino and non-Hispanic or non-Latino populations.^17,18,19,20^ This study addressed this gap by analyzing data from a large, nationally representative sample that included a broader spectrum of racial and ethnic groups, including Asian and multiracial populations, who are often underrepresented in health research or aggregated into broad categories that mask subgroup differences.^24,25,26^

Using cross-sectional data from the 2023 US National Health Interview Survey (NHIS), this study investigated associations between discrimination and mental health among US adults. We hypothesized that higher exposure to discrimination is associated with increased odds of positive screening results for depression and anxiety. We also hypothesized that the associations between discrimination and mental health would differ based on gender and race and ethnicity, with women and individuals of racial and ethnic minority groups experiencing greater odds of depression and anxiety with increasing levels of discrimination. (We use the term *gender* in the Introduction and Discussion to capture the social, cultural, environmental, and behavioral factors that shape gender identity and health. We use the term *sex* in the Methods and Results to refer to the measurement of biological sex in the data.^27^)

## Methods

### Data Source and Study Population

This cross-sectional study used data from the NHIS, an annual survey conducted by the National Center for Health Statistics (NCHS) that uses a multistage probability sampling design to obtain a representative sample of the US population. In 2023, the NCHS sampled and interviewed 29 522 adults aged 18 years or older living in 50 US states and the District of Columbia; this sample was weighted to represent 258 237 552 civilian, noninstitutionalized US adults. Additional survey design and sampling details are available through the NCHS. The study was determined not to involve human participants and was exempted by the the Brown University Health institutional review board. Informed consent was waived because data were deidentified and had been previously collected. This study followed the Strengthening the Reporting of Observational Studies in Epidemiology (STROBE) reporting guideline for reporting cross-sectional data.^28^

### Exposure Measures

Exposure to discrimination was assessed using the following questions from the 5-item Everyday Discrimination Scale^8^: (1) “How often are you treated with less courtesy or respect than others?” (2) “How often do you receive poorer service at restaurants or stores compared to others?” (3) “How often do people act as if they think you are not smart?” (4) “How often do people act as if they are afraid of you?” (5) “How often are you threatened or harassed?” Responses were categorized as: 0, never; 1, less than once a year; 2, a few times a year; 3, a few times a month; and 4, at least once a week. The computed scale’s reliability coefficient (Cronbach α) was 0.73 in this study and 0.77 in prior research,^29^ demonstrating acceptable internal consistency.^30^ A summative scale, ranging from 0 to 20, was constructed by aggregating response scores across scale items, with higher scores indicating greater discrimination. Factor analysis confirmed the scale’s unidimensionality. Discrimination scores were categorized as none (0), low (1-10), and high (11-20) and analyzed continuously to assess degree of exposure and nominally to compare low and high levels of exposure with no exposure.

### Outcome Measures

Depression was assessed using the Patient Health Questionnaire–2 (PHQ-2) scale, a subset of the PHQ-9 scale,^31^ and consisted of the following 2 items: (1) frequency of little interest or pleasure in doing things, and (2) frequency of feeling down, depressed, or hopeless over the past 2 weeks. Response categories were: not at all, several days, more than half the days, or nearly every day. Scores were categorized as negative (0-2) or positive (3-6), with the PHQ-2 having 83% sensitivity and 92% specificity for major depression.^32^

Anxiety was evaluated using the Generalized Anxiety Disorder–2 (GAD-2) scale, a subset of the GAD-7 scale,^33^ and included the following 2 items: (1) frequency of feeling nervous, anxious, or on edge; and (2) frequency of being unable to stop worrying over the past 2 weeks. Responses were the same as the PHQ-2; scores were similarly categorized as negative (0-2) or positive (3-6), with the GAD-2 having 86% sensitivity and 83% specificity for generalized anxiety disorder.^34^ The GAD-2 and the PHQ-2 serve as screening tools for further evaluation rather than diagnostic instruments.^35^ Given the comorbidity of depression and anxiety,^36^ we created a nominal outcome variable with the following 4 categories: 0, negative for both anxiety and depression; 1, positive for anxiety only; 2, positive for depression only; and 3, positive for both depression and anxiety.

### Covariates

Covariates of interest included: age, marital status, nativity (US-born or not), language spoken at home (English, Spanish, other), family structure (number of children), measures of socioeconomic status (educational level, employment status, federal poverty level, food security status, and health insurance coverage), self-reported health status (general health, number of chronic conditions, disability status, and body mass index–based weight status), and place of residence (metropolitan size and regions). Sex and race and ethnicity were also considered when not examined as effect modifiers.

### Effect Modifiers

Self-reported race and ethnicity (Hispanic or Latino, non-Hispanic or non-Latino Asian [hereafter, *Asian*], non-Hispanic or non-Latino Black [hereafter, *Black*], non-Hispanic or non-Latino White [hereafter, *White*], and multiracial or other [non-Hispanic or non-Latino American Indian and Alaska Native only; non-Hispanic or non-Latino American Indian and Alaska Native and any other group; other single and multiple races]) and biological sex (male or female) were examined as effect modifiers.

### Statistical Analysis

All descriptive and regression analyses were adjusted for the NHIS complex survey design (ie, sampling weights, primary sampling units, and stratification included in the dataset) to obtain population estimates.^37^ Statistical tests were 2-sided, with significance a priori determined at an α level of .05. Analyses were performed with STATA/MP, version 18.0 (StataCorp LLC).^38^

Descriptive statistics on exposures, outcomes, and covariates were examined by level of discrimination. The Rao-Scott χ^2^ test of independence and the Wald test were used to determine statistical significance between groups. Prior to conducting regression models, multicollinearity among variables based on variance inflator factors was assessed. The mean variance inflator factor was 1.34 (minimum, 1.03; maximum, 2.05), indicating relatively low multicollinearity.^39,40^

To test the hypothesis that discrimination is positively associated with odds of positive screening results for depression and anxiety, we examined the independent association between the main exposure of interest (discrimination on a continuous and nominal measurement scale) and each outcome of interest, controlling for covariates using multinomial logistic regression models. To test our hypotheses that associations between discrimination and mental health vary by race and ethnicity and sex, we estimated multinomial logistic regression models using a 2-way interaction between discrimination and race and ethnicity and between discrimination and sex. Due to the complexity of interpreting product terms in nonlinear models,^41,42,43,44^ a post hoc design–adjusted Wald test was performed to test our hypothesis. Probabilities were plotted to aid in the interpretation of these multiplicative interaction estimates.

## Results

### Descriptive Analysis

In 2023, data from 29 522 NHIS adult respondents were weighted to represent a population of 258 237 552 civilian, noninstitutionalized US adults (mean age, 48.1 years [95% CI, 47.8-48.4 years]; 51.1% female and 48.9% male; 17.5% Hispanic or Latino, 2.5% multiracial or other, 6.2% non-Hispanic or non-Latino Asian, 11.6% non-Hispanic or non-Latino Black, and 62.2% non-Hispanic or non-Latino White) (Table 1).^45,46^ Over half the US population (55.8%) reported experiencing some level of discrimination, with 52.2% scoring low (1-10) and 3.6% scoring high (11-20). Among racial and ethnic groups, high discrimination scores were highest among Black adults (8.6%), followed by multiracial or other adults (6.4%), Hispanic or Latino adults (3.1%), White adults (2.9%), and Asian adults (2.1%) (*P* < .001).

**Table 1.** Describing Discrimination Across Anxiety and Depressive Symptoms, Sociodemographic Characteristics, and Health Characteristics of US Civilian Adults Aged 18 Years or Older^a^

Characteristic	Everyday Discrimination Scale scores			Total study sample	*P* value^b^
	None (0)	Low (1-10)	High (11-20)		
Weighted % (95% CI)	44.2 (43.2-45.2)	52.2 (51.3-53.1)	3.6 (3.3-3.9)	100.0	NA
Outcome, % (95% CI)					
Negative screening result for depression and anxiety	47.2 (46.1-48.2)	50.5 (49.5-51.5)	2.4 (2.1-2.6)	88.0 (87.5-88.5)	<.001
Positive screening result for anxiety	23.5 (20.8-26.4)	66.8 (63.7-69.8)	9.7 (7.8-12.1)	5.0 (4.8-5.4)	
Positive screening result for depression	25.9 (22.5-29.7)	63.5 (59.4-67.4)	10.6 (8.1-13.7)	2.7 (2.5-2.9)	
Positive screening result for both depression and anxiety	18.7 (16.1-21.6)	63.6 (60.2-66.9)	17.7 (15.1-20.6)	4.2 (4.0-4.5)	
Characteristics					
Age, mean (95% CI)	51.2 (50.8-51.7)	45.9 (45.6-46.3)	40.1 (38.8-41.3)	48.1 (47.8-48.4)	<.001
Biological sex, % (95% CI)					
Male	45.4 (44.1-46.6)	50.9 (49.7-52.1)	3.7 (3.3-4.1)	48.9 (48.2-49.6)	.003
Female	43.1 (41.9-44.2)	53.4 (52.3-54.6)	3.5 (3.1-3.9)	51.1 (50.4-51.8)	
Race and ethnicity, % (95% CI)					
Asian	47.8 (44.5-51.0)	50.2 (46.9-53.4)	2.1 (1.4-3.0)	6.2 (5.7-6.8)	<.001
Black	32.6 (30.3-35.0)	58.8 (56.4-61.1)	8.6 (7.4-10.1)	11.6 (10.7-12.4)	
Hispanic or Latino	52.3 (50.1-54.4)	44.6 (42.6-46.7)	3.1 (2.6-3.7)	17.5 (16.3-18.8)	
White	44.1 (43.1-45.2)	53.0 (51.9-54.1)	2.9 (2.6-3.2)	62.2 (60.8-63.7)	
Multiracial or other^c^^,^^d^	34.1 (29.5-38.9)	59.6 (54.8-64.2)	6.4 (4.6-8.7)	2.5 (2.2-2.9)	
Language spoken at home, % (95% CI)					
English	42.6 (41.6-43.6)	53.6 (52.7-54.6)	3.8 (3.5-4.1)	83.3 (82.3-84.3)	<.001
Spanish	54.4 (51.7-57.0)	42.7 (40.1-45.4)	2.9 (2.2-3.8)	10.4 (9.6-11.3)	
Other	48.5 (45.5-51.6)	48.9 (46.0-51.8)	2.6 (1.7-3.8)	6.3 (5.8-6.8)	
Marital status, % (95% CI)^c^					
Single, divorced, widowed, or separated	42.0 (40.7-43.3)	53.0 (51.8-54.2)	5.0 (4.5-5.6)	40.8 (40.0-41.6)	<.001
Married or partnered	45.7 (44.6-46.8)	51.7 (50.5-52.8)	2.7 (2.4-3.0)	59.2 (58.4-60.0)	
Federal poverty level, % (95% CI)^c^					
<100%	46.7 (44.3-49.1)	47.0 (44.7-49.3)	6.3 (5.3-7.5)	10.0 (9.4-10.6)	<.001
100%-199%	47.5 (45.8-49.3)	48.3 (46.6-50.0)	4.2 (3.5-5.0)	18.0 (17.4-18.7)	
200%-299%	45.1 (43.2-46.9)	50.7 (48.8-52.5)	4.2 (3.6-5.1)	17.1 (16.5-17.7)	
300%-399%	44.0 (41.9-46.1)	52.8 (50.7-54.9)	3.2 (2.6-3.9)	12.9 (12.5-13.4)	
400%-499%	40.3 (38.2-42.5)	56.6 (54.4-58.7)	3.1 (2.4-3.9)	10.5 (10.0-10.9)	
500%-599%	42.3 (41.0-43.6)	55.2 (53.9-56.5)	2.5 (2.1-2.9)	31.5 (30.5-32.6)	
Educational level, % (95% CI)^c^					
<Bachelor’s degree	45.8 (44.6-47.0)	50.0 (48.8-51.1)	4.2 (3.8-4.7)	66.8 (65.8-67.7)	<.001
≥Bachelor’s degree	40.7 (39.5-42.0)	56.9 (55.7-58.2)	2.4 (2.0-2.7)	33.2 (32.3-34.2)	
Employment status, % (95% CI)^c^					
Worked for pay last wk	39.0 (37.8-40.2)	57.1 (55.9-58.2)	3.9 (3.6-4.4)	62.5 (61.7-63.2)	<.001
Worked for pay within past 12 mo	39.9 (37.1-42.8)	55.7 (52.8-58.6)	4.4 (3.3-5.9)	6.5 (6.2-6.9)	
Worked for pay 1-5 years ago	49.1 (46.8-51.5)	47.7 (45.3-50.1)	3.2 (2.4-4.1)	8.9 (8.5-9.3)	
Worked for pay >5 years ago	57.8 (56.4-59.2)	40.0 (38.6-41.3)	2.2 (1.8-2.8)	19.4 (18.9-20.0)	
Never worked for pay	58.8 (53.2-64.2)	37.0 (32.0-42.4)	4.2 (2.4-7.0)	2.7 (2.4-3.0)	
Nativity, born in the US, % (95% CI)					
No	54.9 (52.8-57.0)	43.0 (41.0-45.1)	2.1 (1.6-2.6)	19.0 (18.0-20.0)	<.001
Yes	41.6 (40.6-42.6)	54.4 (53.5-55.4)	3.9 (3.6-4.3)	81.0 (80.0-82.0)	
No. of children in family, % (95% CI)					
0	44.3 (43.3-45.4)	52.0 (51.0-53.1)	3.6 (3.3-4.0)	67.9 (67.1-68.7)	<.001
1	41.5 (39.3-43.8)	54.1 (51.8-56.3)	4.4 (3.6-5.3)	14.2 (13.6-14.8)	
2	43.6 (41.4-45.9)	53.6 (51.4-55.9)	2.7 (2.2-3.5)	11.3 (10.9-11.8)	
≥3	49.4 (46.4-52.4)	47.2 (44.2-50.2)	3.4 (2.5-4.6)	6.6 (6.2-7.0)	
Food security status, % (95% CI)					
Food secure	45.7 (44.7-46.7)	51.6 (50.6-52.6)	2.7 (2.5-3.0)	91.0 (90.5-91.5)	<.001
Low food security	33.0 (29.7-36.5)	58.3 (54.8-61.7)	8.7 (7.0-10.8)	5.1 (4.8-5.5)	
Very low food security	22.5 (19.5-25.8)	60.0 (56.2-63.7)	17.5 (14.7-20.8)	3.9 (3.6-4.2)	
Health insurance coverage, % (95% CI)					
No	47.1 (44.0-50.2)	47.8 (44.8-50.9)	5.1 (4.0-6.5)	7.4 (6.9-8.0)	<.001
Yes	44.0 (43.0-45.0)	52.6 (51.6-53.5)	3.5 (3.2-3.8)	92.6 (92.0-93.1)	
Metropolitan size, % (95% CI)					
Large central metropolitan	43.8 (42.1-45.5)	52.2 (50.6-53.7)	4.1 (3.5-4.6)	30.3 (28.3-32.5)	<.001
Large fringe metropolitan	42.6 (41.0-44.1)	54.4 (52.8-55.9)	3.1 (2.6-3.7)	25.4 (23.2-27.8)	
Medium and small metropolitan	43.5 (41.8-45.3)	52.5 (50.8-54.2)	4.0 (3.4-4.6)	30.4 (27.7-33.2)	
Nonmetropolitan	49.5 (46.4-52.6)	47.6 (44.7-50.6)	2.9 (2.2-3.7)	13.9 (13.0-14.7)	
Region, % (95% CI)					
Northeast	47.8 (45.3-50.4)	49.5 (47.1-52.0)	2.6 (2.1-3.3)	17.2 (16.2-18.2)	<.001
Midwest	41.9 (40.1-43.7)	54.2 (52.5-55.9)	3.9 (3.3-4.6)	20.7 (19.7-21.7)	
South	44.3 (42.6-46.0)	51.7 (50.1-53.3)	4.0 (3.5-4.5)	38.5 (37.0-40.0)	
West	43.4 (41.4-45.4)	53.2 (51.2-55.1)	3.5 (2.9-4.2)	23.7 (22.4-24.9)	
Health status, % (95% CI)					
Excellent	52.1 (50.3-53.9)	45.7 (44.0-47.4)	2.2 (1.8-2.8)	22.1 (21.5-22.8)	<.001
Very good	41.7 (40.4-43.1)	55.4 (54.0-56.7)	2.9 (2.5-3.4)	33.7 (33.0-34.5)	
Good	42.9 (41.4-44.3)	53.2 (51.8-54.6)	3.9 (3.4-4.5)	29.1 (28.5-29.8)	
Fair	40.5 (38.3-42.8)	53.3 (51.1-55.4)	6.2 (5.2-7.3)	11.7 (11.2-12.2)	
Poor	41.0 (37.4-44.6)	50.8 (47.0-54.5)	8.2 (6.4-10.5)	3.3 (3.1-3.6)	
Disability status, % (95% CI)					
No	44.5 (43.5-45.5)	52.3 (51.3-53.3)	3.2 (2.9-3.4)	90.7 (90.3-91.1)	<.001
Yes	41.0 (38.9-43.2)	50.9 (48.7-53.1)	8.1 (6.8-9.5)	9.3 (8.9-9.7)	
No. of chronic conditions, % (95% CI)^c^					
0	44.2 (42.8-45.5)	52.7 (51.4-54.0)	3.2 (2.8-3.6)	45.4 (44.6-46.2)	<.001
1	42.8 (41.4-44.2)	53.4 (52.0-54.8)	3.8 (3.3-4.4)	27.5 (26.9-28.1)	
≥2	45.7 (44.2-47.1)	50.2 (48.8-51.6)	4.1 (3.6-4.7)	27.1 (26.5-27.8)	
Body mass index status, % (95% CI)					
Underweight	45.7 (39.8-51.8)	49.4 (43.5-55.4)	4.8 (2.8-8.4)	1.6 (1.4-1.8)	<.001
Healthy weight	44.5 (43.1-45.9)	52.3 (50.9-53.6)	3.2 (2.8-3.8)	30.5 (29.8-31.2)	
Overweight	46.9 (45.5-48.4)	49.9 (48.5-51.3)	3.1 (2.7-3.6)	33.9 (33.3-34.6)	
Obese	40.6 (39.1-42.0)	55.1 (53.7-56.5)	4.3 (3.8-4.9)	34.0 (33.2-34.7)	

Abbreviation: NA, not applicable.

^a^ Source: 2023 US National Health Interview Survey—National Center for Health Statistics (NCHS), Centers for Disease Control and Prevention.^45^ Unweighted n = 29 522; weighted to represent a population of 258 237 552 noninstitutionalized civilian US adults. Valid row percentages and 95% CIs are weighted to represent population estimates. The number of diagnosed chronic conditions included stroke, cancer, asthma, diabetes, arthritis, chronic obstructive pulmonary disease, hypertension, coronary heart disease, and hepatitis. Disability status is derived from the Washington Group Short Set Composite Disability Indicator computed by the NCHS.^46^

^b^ The statistical significance for continuous age is based on the Adjusted Wald test and the Rao-Scott χ^2^ test of independence for categorical variables.

^c^ Recombined categories.

^d^ Non-Hispanic or non-Latino American Indian and Alaska Native only; non-Hispanic or non-Latino American Indian and Alaska Native and any other group; other single and multiple races.

Exposure to discrimination also varied significantly across several other sociodemographic characteristics and health indicators (Table 1).^45,46^ Higher percentages of females, those born outside the US, individuals experiencing food insecurity, those with disabilities, and those with obesity reported some level of discrimination compared with their counterparts. A higher percentage of adults with a positive screening result for depression (10.6%), anxiety (9.7%), or both (17.7%) reported high exposure to discrimination compared with those with a negative screening result (2.4%) (*P* < .001).

### Discrimination and Odds of Depression and Anxiety

Results from adjusted multinomial logistic regression models indicated that higher levels of discrimination were significantly associated with increased odds of positive screening results for depression, anxiety, and depression and anxiety combined (Table 2).^45^ For every unit-score increase in discrimination, odds of positive screening results increased by 15% for depression (odds ratio [OR], 1.15; [95% CI, 1.12-1.17]; *P* < .001), increased by 14% for anxiety (OR, 1.14; [95% CI, 1.12-1.16]; *P* < .001), or increased by 19% for both (OR, 1.19 [95% CI, 1.16-1.21]; *P* < .001) vs negative screening results for both.

**Table 2.** Adjusted Associations Between Discrimination Scores (Continuous) and Mental Health Screening Among US Civilian Adults: Results From Multinomial Logistic Regression^a^

Outcome	Everyday Discrimination Scale^b^	
	Odds ratio (95% CI)	*P* value
PHQ-2 and GAD-2: negative screening result	1 [Reference]	NA
GAD-2: positive screening result	1.14 (1.12-1.16)	<.001
PHQ-2: positive screening result	1.15 (1.12-1.17)	<.001
PHQ-2 and GAD-2: positive screening result	1.19 (1.16-1.27)	<.001

Abbreviations: GAD-2, Generalized Anxiety Disorder–2 scale; NA, not applicable; PHQ-2, Patient Health Questionnaire–2 scale.

^a^ Source: 2023 US National Health Interview Survey—National Center for Health Statistics (NCHS), Centers for Disease Control and Prevention.^45^

^b^ The scale is continuous (range, 0-20). Regression models adjusted for age, age^2^ (continuous age was squared to capture nonlinearity between outcomes and age), sex, race and ethnicity, language spoken at home, nativity, marital status, educational level, federal poverty level, employment status, food security status, number of children in the family, metropolitan size (based on the 2013 NCHS Urban-Rural Classification Scheme for Counties), region of residence, self-reported health status, number of chronic conditions, and body mass index categories.

Table 3^45^ presents adjusted multinomial logistic regression models estimating odds of positive screening results on mental health tests comparing those reporting high or low levels of discrimination with those reporting no discrimination. Adults with high levels of discrimination had significantly greater odds of positive screening results for depression (OR, 5.39 [95% CI, 3.61-8.04]; *P* < .001), anxiety (OR, 4.98 [95% CI, 3.59-6.91]; *P* < .001), and both depression and anxiety (OR, 8.84 [95% CI, 6.44-12.14]; *P* < .001) compared with those not exposed to discrimination. Adults with low levels of discrimination also had higher odds, with approximately twice the odds of positive screening results for depression (OR, 2.20 [95% CI, 1.77-2.72]; *P* < .001), anxiety (OR, 1.97 [95% CI, 1.66-2.33]; *P* < .001), and both depression and anxiety (OR, 2.60 [95% CI, 2.13-3.18]; *P* < .001) compared with those not exposed.

**Table 3.** Adjusted Associations Between Exposure to Discrimination and Mental Health Screening Among US Civilian Adults: Results From Multinomial Logistic Regression^a^

Outcome	Everyday Discrimination Scale^b^			
	Odds ratio (95% CI)			*P* value
	Score 0 (reference exposure)	Scores 1-10 (low)	Scores 11-20 (high)	
PHQ-2 and GAD-2: negative screening result	1 [Reference]	1 [Reference]	1 [Reference]	NA
GAD-2: positive screening result	1 [Reference]	1.97 (1.66-2.33)	4.98 (3.59-6.91)	<.001
PHQ-2: positive screening result	1 [Reference]	2.20 (1.77-2.72)	5.39 (3.61-8.04)	<.001
PHQ-2 and GAD-2: positive screening result	1 [Reference]	2.60 (2.13-3.18)	8.84 (6.44-12.14)	<.001

Abbreviations: GAD-2, Generalized Anxiety Disorder–2 scale; NA, not applicable; PHQ-2, Patient Health Questionnaire–2 scale.

^a^ Source: 2023 US National Health Interview Survey—National Center for Health Statistics (NCHS), Centers for Disease Control and Prevention.^45^

^b^ Exposure to discrimination is polytomous. Regression models adjusted for age, age^2^ (continuous age was squared to capture nonlinearity between outcomes and age), sex, race and ethnicity, language spoken at home, nativity, marital status, educational level, federal poverty level, employment status, food security status, number of children in the family, metropolitan size (based on the 2013 NCHS Urban-Rural Classification Scheme for Counties), region of residence, self-reported health status, number of chronic conditions, and body mass index categories.

### Effect Modification by Race and Ethnicity and Sex

**Figure 1.**
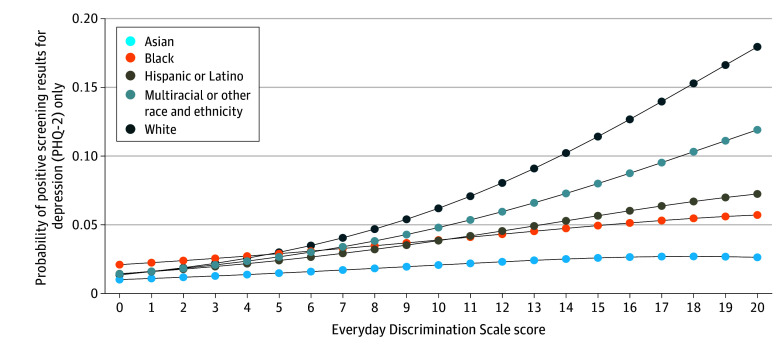
Adjusted Association Between Discrimination and Positive Screening Results for Depression: the Moderating Association of Race and Ethnicity Adjusted Wald test: *F*_4,607_ = 3.35; *P* = .01. Associations adjusted for age, age^2^ (continuous age was squared to capture nonlinearity between outcomes and age), sex, language spoken at home, nativity, marital status, educational level, federal poverty level, employment status, food security status, number of children in the family, metropolitan size, region of residence, self-reported health status, number of chronic conditions, and body mass index categories. PHQ-2 indicates Patient Health Questionnaire–2 scale.

**Figure 2.**
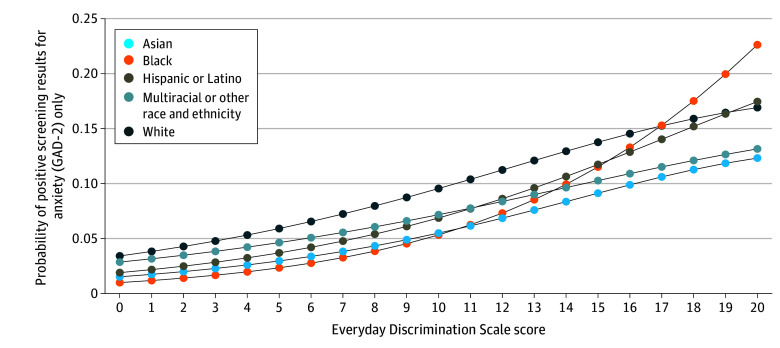
Adjusted Association Between Discrimination and Positive Screening Results for Anxiety: the Moderating Association of Race and Ethnicity Adjusted Wald test: *F*_4,607_ = 1.06; *P* = .38. Associations adjusted for age, age^2^ (continuous age was squared to capture nonlinearity between outcomes and age), sex, language spoken at home, nativity, marital status, educational level, federal poverty level, employment status, food security status, number of children in the family, metropolitan size, region of residence, self-reported health status, number of chronic conditions, and body mass index categories. GAD-2 indicates Generalized Anxiety Disorder–2 scale.

Race and ethnicity was a significant moderator of the association between discrimination and positive screening results for depression alone (*F*_4,607_ = 3.35; *P* = .01) and positive screening results for both depression and anxiety (*F*_4,607_ = 2.80; *P* = .03). As exposure to discrimination increased, the probability of positive screening results for depression varied by race and ethnicity, increasing more sharply among White and multiracial or other race adults (Figure 1). Race and ethnicity was not a significant moderator of the association between discrimination and positive screening results for anxiety (*F*_4,607_ = 1.06; *P* = .38) (Figure 2). Similarly, higher levels of discrimination corresponded with varying probabilities of positive screening results for both depression and anxiety depending on race and ethnicity, with a steeper increase observed among Asian adults (eFigure 1 in Supplement 1). Sex was not a moderator of the association between discrimination and positive screening results for depression (*F*_1,610_ = 0.53; *P* = .47), anxiety (*F*_1,610_ = 0.00, *P* = .99), or both (*F*_1,610_ = 2.07, *P* = .15) (eFigures 2-4 in Supplement 1).

## Discussion

This study reveals critical insights into the associations between discrimination, depression, and anxiety among US adults. In 2023, over half of US adults reported experiencing some level of discrimination, with higher rates among women than men and among Black and multiracial or other race adults than individuals of other racial or ethnic groups. Higher percentages of adults born outside the US and those with socioeconomic or health disadvantages (eg, those experiencing food insecurity, those with disabilities) also reported some level of discrimination compared with their counterparts.

Our findings demonstrate that greater exposure to discrimination was associated with increased odds of positive screening results for depression, anxiety, and both depression and anxiety across the general US adult population and within all gender and racial and ethnic groups. With increasing levels of discrimination, the odds of depression and anxiety increased. Adults with high exposure to discrimination had the highest odds of positive screening results for adverse mental health, with 5 times the odds of positive screening results for depression and anxiety, and nearly 9 times the odds of positive screening results for both depression and anxiety than adults reporting no exposure to discrimination.

These results align with cross-sectional and longitudinal research demonstrating that experiencing discrimination is associated with psychological distress,^1^ depression, and anxiety.^6,17,18^ However, prior research on the association between discrimination and mental health has often been limited by smaller sample sizes or focused primarily on comparisons between Black and White populations or between Hispanic or Latino and non-Hispanic or non-Latino populations.^17,18,19,20^ By analyzing data from a large, nationally representative sample that encompasses a wider range of racial and ethnic groups, including Asian and multiracial populations, who are often underrepresented or aggregated in health research,^24,25,26^ this study expands beyond comparisons between Black and White populations and highlights the association between discrimination and mental health across the US adult population.

Our study further revealed that increased probabilities of depression and anxiety based on exposure to discrimination varied by race and ethnicity. As exposure to discrimination increased, the probability of positive screening results for depression varied depending on race and ethnicity, with a more pronounced increase observed among multiracial and other race adults and White adults. High levels of discrimination were also associated with differing probabilities of positive screening results for both anxiety and depression across racial and ethnic groups, with a more significant increase noted among Asian adults. The increased probability of positive screening results for anxiety based on discrimination levels was consistent across race and ethnicity.

The finding that the association between discrimination and mental health differs by race and ethnicity, with greater probability of depression and anxiety among multiracial and other race adults, White adults, and Asian adults, may be associated with a combination of social, cultural, and systemic factors. For multiracial individuals, experiences of discrimination and its association with mental health can be multifaceted due to challenges of navigating multiple racial identities across their lifecourse, which may intensify feelings of isolation.^47^ The association between poorer mental health and being multiracial is complex, involving social and structural determinants as well as individual risk behaviors (eg, substance use) and exposure to stress.^48^ This complexity may heighten the psychological effect of discrimination, leading to more pronounced depressive symptoms and poorer mental health compared with other racial and ethnic groups.^49^ For White individuals, the association of different forms of discrimination with mental health is less well understood. One potential explanation is that while White adults generally report lower levels of race- and ethnicity-based discrimination compared with other racial and ethnic groups, they may experience other forms of mistreatment based on factors such as income or educational level.^50^

Asian adults may experience a sharper increase in depression and anxiety with higher exposure to discrimination due to unique societal stressors.^51^ “Model minority” stereotyping (the assumption that all Asians are successful, hardworking, and high-achieving) can lead to unrealistic expectations and pressure, as well as overlook the social and economic struggles faced by individuals within this group.^52^ In addition, discrimination related to language and stereotypes of Asians as “perpetual foreigners” can affect job opportunities, social integration, and access to services that further compound stress faced by Asian communities.^53^ Studies show that anti-Asian racism increased during the COVID-19 pandemic and was associated with adverse mental health among Asian individuals in the US.^54^ Combined with cultural stigma around mental health,^55^ the steeper increase in risk of depression and anxiety among Asian adults may reflect the cumulative impact of these multiple, intersecting pressures.

The racial and ethnic differences observed in our study contribute to the growing body of evidence demonstrating that marginalized racial and ethnic groups generally face higher levels of discrimination and associated mental health challenges than White peers^1,2,8,56,57^ and warrant deeper investigation. Study findings should not diminish the profound and well-documented experiences of discrimination among Black, Hispanic or Latino, and members of other marginalized racial and ethnic populations,^1,2^ who have long faced and continue to experience systemic and structural racism that significantly affects their mental and physical health.^5^ Although our study’s results demonstrate that White adults experienced higher odds of positive screening results for depression with increasing levels of discrimination, this finding does not imply that exposure to discrimination is less significant for Asian, Black, Hispanic or Latino, and other racial and ethnic populations. One potential explanation is that, after centuries of systemic racism and discrimination, individuals in these groups may have developed a form of resignation, passive acceptance, or normalization of these experiences, where prolonged exposure to discrimination may lead some groups to internalize its effects or develop coping mechanisms that mitigate the perceived effect.^58^ Exploring these dynamics further could help improve our understanding of how discrimination is associated with mental health outcomes across different racial and ethnic groups.

Our study showed that the negative association of discrimination with anxiety and depression was similar for men and women. This finding may indicate that both genders experience similar levels of distress, coping challenges, and psychological responses to discriminatory experiences. This finding also suggests the need to consider other factors, such as the form of discrimination and its cumulative or intersectional effect.^59,60^

Findings underscore the need for ongoing research into the long-term mental health effects of discrimination across diverse populations. Future studies can further investigate how various forms of discrimination, such as those based on socioeconomic status and preferred language, are uniquely associated with mental health, especially among marginalized groups and those with greater mental health burdens. Expanding access to mental health services and insurance coverage remains essential for equitable care.^61,62^ Targeted mental health support programs can be enhanced to acknowledge the distinct experiences, stressors, and cultural factors faced by different racial and ethnic groups,^63^ recognizing that the association between discrimination and mental health may vary across populations.

### Limitations

This study has some limitations, including the cross-sectional design, which limits our ability to infer directionality between exposure and outcome. A bidirectional association is possible, where individuals with existing depression or anxiety may experience discrimination more negatively, potentially influencing study findings. Self-reported measures may be subject to recall bias, and discrimination may be underreported or overreported. The Everyday Discrimination Scale measures general discrimination without specifying type (eg, race and ethnicity, gender, socioeconomic status, LGBTQ+ status), context, or setting, limiting our ability to identify which forms are most associated with mental health outcomes. Socioeconomic and cultural factors influencing the association between discrimination and mental health were not fully explored. Addressing these limitations in future research could enhance the understanding of these complex associations and guide intervention strategies.

## Conclusions

The findings of this cross-sectional study highlight a significant association between discrimination, depression, and anxiety among US adults, underscoring the need for further mental health evaluation and greater awareness of how these associations differ across genders and race and ethnicity.

## Notes

Supplement 1. **eFigure 1.** Adjusted Association Between Discrimination and Screening Positive for Depression and Anxiety: the Moderating Effect of Race and Ethnicity **eFigure 2.** Adjusted Association Between Discrimination and Screening Positive for Depression: the Moderating Effect of Biological Sex **eFigure 3.** Adjusted Association Between Discrimination and Screening Positive for Anxiety: the Moderating Effect of Biological Sex **eFigure 4.** Adjusted Association Between Discrimination and Screening Positive for Depression and Anxiety: the Moderating Effect of Biological Sex

Supplement 2.
Data Sharing Statement
