# Supplementary material for: Discrimination, Depression, and Anxiety Among US Adults
Source: JAMA Netw Open. 2025 Mar 28;8(3):e252404. doi: 10.1001/jamanetworkopen.2025.2404 (PMC11953758; doi:10.1001/jamanetworkopen.2025.2404)
Supplement: Supplement 1. — eFigure 1. Adjusted Association Between Discrimination and Screening Positive for Depression and Anxiety: the Moderating Effect of Race and Ethnicity eFigure 2. Adjusted Association Between Discrimination and Screening Positive for Depression: the Moderating Effect of Biological Sex eFigure 3. Adjusted Association Between Discrimination and Screening Positive for Anxiety: the Moderating Effect of Biological Sex eFigure 4. Adjusted Association Between Discrimination and Screening Positive for Depression and Anxiety: the Moderating Effect of Biological Sex [file jamanetwopen-e252404-s001.pdf]

Supplementary Online Content

Wang ML, Narcisse MR. Discrimination, depression, and anxiety among US adults. *JAMA Netw Open.* 2025;8(3):e252404.
doi:10.1001/jamanetworkopen.2025.2404

eFigure 1. Adjusted Association Between Discrimination and Screening Positive for Depression and Anxiety: the Moderating Effect of Race and Ethnicity

eFigure 2. Adjusted Association Between Discrimination and Screening Positive for Depression: the Moderating Effect of Biological Sex

eFigure 3. Adjusted Association Between Discrimination and Screening Positive for Anxiety: the Moderating Effect of Biological Sex

eFigure 4. Adjusted Association Between Discrimination and Screening Positive for Depression and Anxiety: the Moderating Effect of Biological Sex

This supplementary material has been provided by the authors to give readers additional information about their work.

© 2025 Wang ML et al. *JAMA Network Open.*

eFigure 1. Adjusted Association Between Discrimination and Screening Positive for Depression and Anxiety: the Moderating Effect of Race and Ethnicity

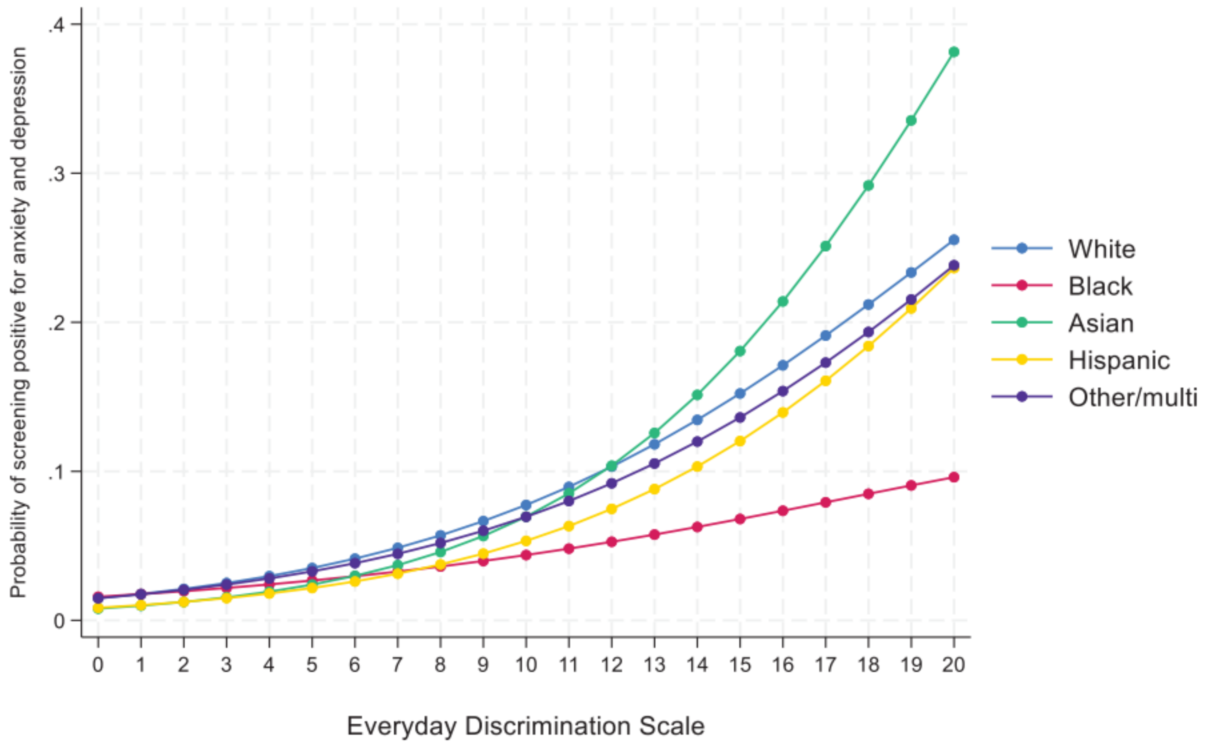

Adjusted Wald Test: ($F_{4,\,607}$=2.80; p=0.03)

Note: Associations adjusted for age, age^2 (continuous age was squared to capture non-linearity between outcomes and age), sex, language spoken at home, nativity, marital status, education, federal poverty level, employment status, food security status, number of children in the family, metropolitan size, region of residence, self-reported health status, number of chronic conditions, and body mass index categories.

© 2025 Wang ML et al. *JAMA Network Open.*

eFigure 2. Adjusted Association Between Discrimination and Screening Positive for Depression: the Moderating Effect of Biological Sex

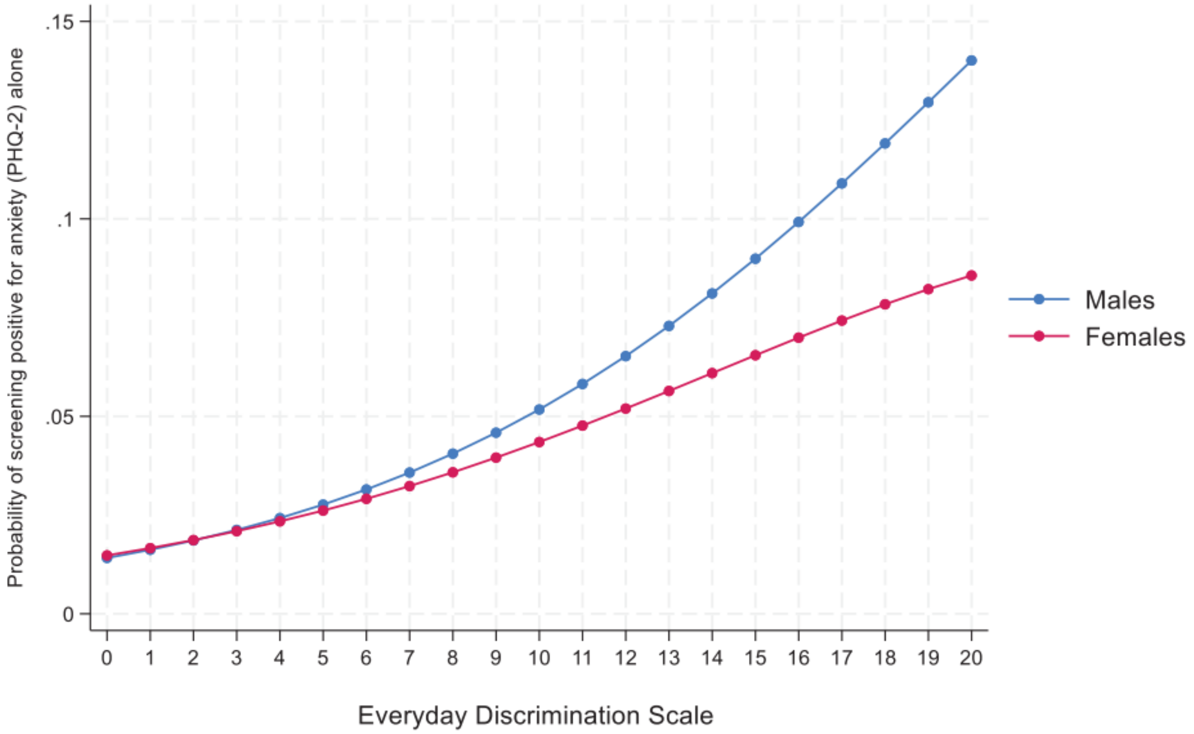

Adjusted Wald Test: ($F_{1,\ 610}$=0.53; p=0.47)

Note: Associations adjusted for age, age^2 (continuous age was squared to capture non-linearity between outcomes and age), race/ethnicity, language spoken at home, nativity, marital status, education, federal poverty level, employment status, food security status, number of children in the family, metropolitan size, region of residence, self-reported health status, number of chronic conditions, and body mass index categories.

© 2025 Wang ML et al. *JAMA Network Open.*

eFigure 3. Adjusted Association Between Discrimination and Screening Positive for Anxiety: the Moderating Effect of Biological Sex

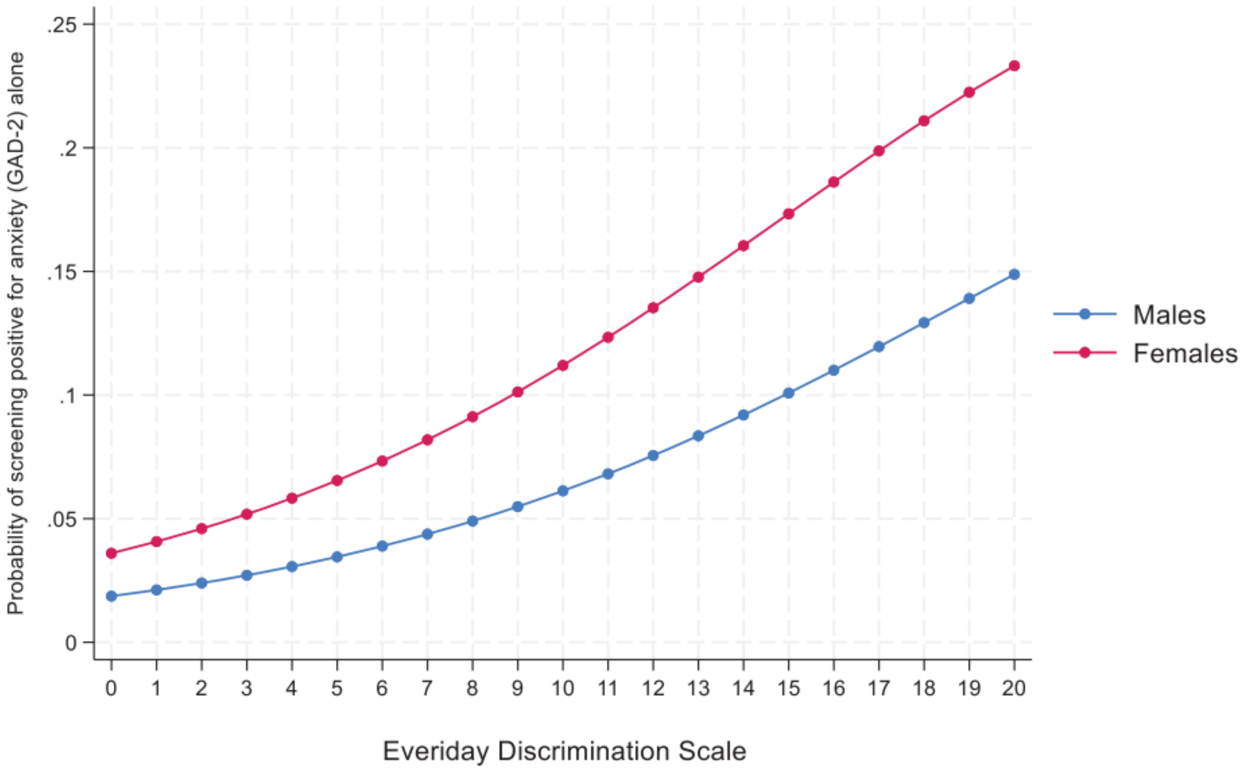

Adjusted Wald Test: ($F_{1, 610}$=0.00, p=0.99)

Note: Associations adjusted for age, age^2 (continuous age was squared to capture non-linearity between outcomes and age), race/ethnicity, language spoken at home, nativity, marital status, education, federal poverty level, employment status, food security status, number of children in the family, metropolitan size, region of residence, self-reported health status, number of chronic conditions, and body mass index categories.

© 2025 Wang ML et al. *JAMA Network Open.*

eFigure 4. Adjusted Association Between Discrimination and Screening Positive for Depression and Anxiety: the Moderating Effect of Biological Sex

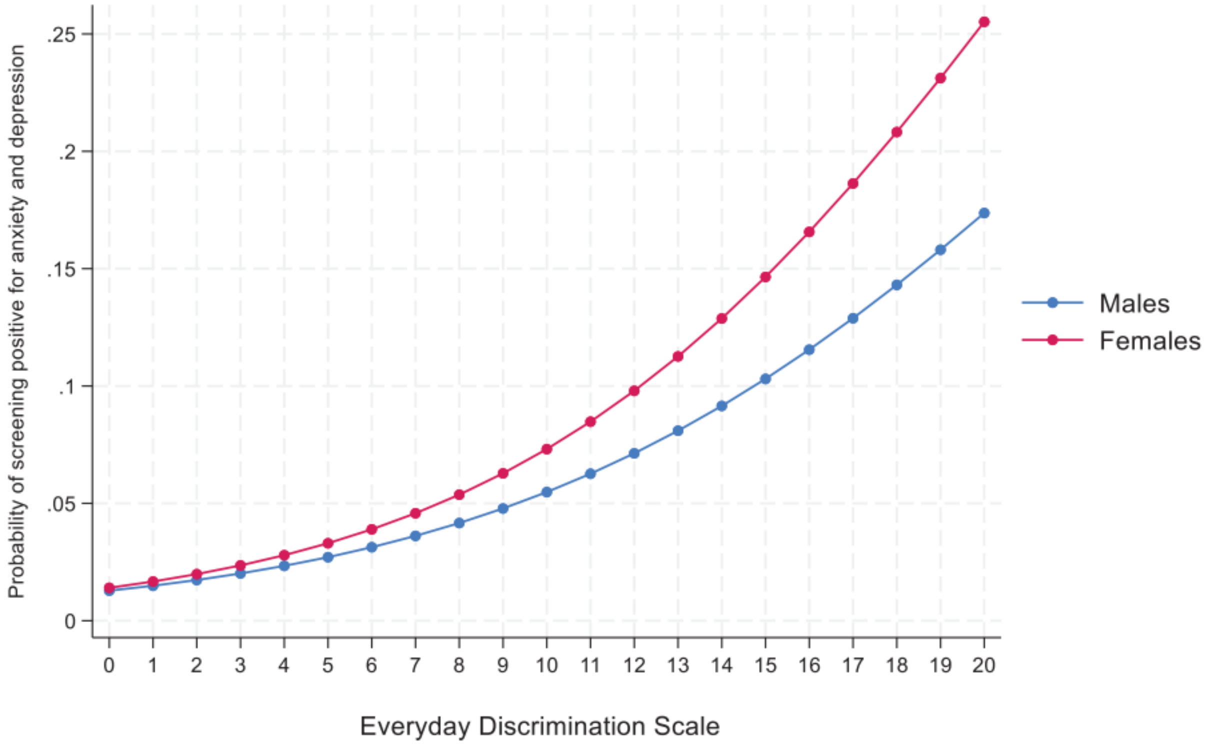

Adjusted Wald Test: ($F_{1,\,610}$=2.07, *p*=0.15)

Note: Associations adjusted for age, age^2 (continuous age was squared to capture non-linearity between outcomes and age), race/ethnicity, language spoken at home, nativity, marital status, education, federal poverty level, employment status, food security status, number of children in the family, metropolitan size, region of residence, self-reported health status, number of chronic conditions, and body mass index categories.

© 2025 Wang ML et al. *JAMA Network Open.*
