# Supplementary material for: Discrimination, Depression, and Anxiety Among US Adults
Source: JAMA Netw Open. 2025 Mar 28;8(3):e252404. doi: 10.1001/jamanetworkopen.2025.2404 (PMC11953758; doi:10.1001/jamanetworkopen.2025.2404)
Supplement: Supplement 2. — Data Sharing Statement [file jamanetwopen-e252404-s002.pdf]

Data Sharing Statement

Wang. Discrimination, Depression, and Anxiety Among US Adults. *JAMA Netw Open*. Published March 28, 2025. doi:10.1001/jamanetworkopen.2025.2404

Data
Data available: Yes
Data types: Other (please specify)
Additional Information: The 2023 National Health Interview Survey (NHIS) is openly made available by the Centers for Diseases Control and Prevention (CDC) at https://www.cdc.gov/nchs/nhis/2023nhis.htm. No datasets were generated for this study. Any analysis, interpretation, and/or conclusion based on the NHIS 2023 data is solely that of the authors. Opinions, conclusions, and recommendations expressed herein do not necessarily represent those of the National Center for Health Statistics or CDC, which are responsible for the data.
How to access data: The 2023 National Health Interview Survey (NHIS) is openly made available by the Centers for Diseases Control and Prevention (CDC) at https://www.cdc.gov/nchs/nhis/2023nhis.htm. No datasets were generated for this study. Any analysis, interpretation, and/or conclusion based on the NHIS 2023 data is solely that of the authors. Opinions, conclusions, and recommendations expressed herein do not necessarily represent those of the National Center for Health Statistics or CDC, which are responsible for the data.
When available: With publication

Supporting Documents
Document types: None

Additional Information
Who can access the data: The 2023 National Health Interview Survey (NHIS) is openly made available by the Centers for Diseases Control and Prevention (CDC) at https://www.cdc.gov/nchs/nhis/2023nhis.htm. No datasets were generated for this study. Any analysis, interpretation, and/or conclusion based on the NHIS 2023 data is solely that of the authors. Opinions, conclusions, and recommendations expressed herein do not necessarily represent those of the National Center for Health Statistics or CDC, which are responsible for the data.
Types of analyses: The 2023 National Health Interview Survey (NHIS) is openly made available by the Centers for Diseases Control and Prevention (CDC) at https://www.cdc.gov/nchs/nhis/2023nhis.htm. No datasets were generated for this study. Any analysis, interpretation, and/or conclusion based on the NHIS 2023 data is solely that of the authors. Opinions, conclusions, and recommendations expressed herein do not necessarily represent those of the National Center for Health Statistics or CDC, which are responsible for the data.
Mechanisms of data availability: The 2023 National Health Interview Survey (NHIS) is openly made available by the Centers for Diseases Control and Prevention (CDC) at https://www.cdc.gov/nchs/nhis/2023nhis.htm. No datasets were generated for this study. Any analysis, interpretation, and/or conclusion based on the NHIS 2023 data is solely that of the authors. Opinions, conclusions, and recommendations expressed herein do not necessarily represent those of the National Center for Health Statistics or CDC, which are responsible for the data.
